# Has Human Evolution Stopped?

**DOI:** 10.5041/RMMJ.10006

**Published:** 2010-07-02

**Authors:** Alan R. Templeton

**Affiliations:** Departments of Biology and Genetics, Washington University, St. Louis, Missouri, USA; Department of Evolutionary and Environmental Biology, University of Haifa, Israel

**Keywords:** human evolution, natural selection, genetic disease, systemic disease, adaptation, cultural evolution

## Abstract

It has been argued that human evolution has stopped because humans now adapt to their environment via cultural evolution and not biological evolution. However, all organisms adapt to their environment, and humans are no exception. Culture defines much of the human environment, so cultural evolution has actually led to adaptive evolution in humans. Examples are given to illustrate the rapid pace of adaptive evolution in response to cultural innovations. These adaptive responses have important implications for infectious diseases, Mendelian genetic diseases, and systemic diseases in current human populations. Moreover, evolution proceeds by mechanisms other than natural selection. The recent growth in human population size has greatly increased the reservoir of mutational variants in the human gene pool, thereby enhancing the potential for human evolution. The increase in human population size coupled with our increased capacity to move across the globe has induced a rapid and ongoing evolutionary shift in how genetic variation is distributed within and among local human populations. In particular, genetic differences between human populations are rapidly diminishing and individual heterozygosity is increasing, with beneficial health effects. Finally, even when cultural evolution eliminates selection on a trait, the trait can still evolve due to natural selection on other traits. Our traits are not isolated, independent units, but rather are integrated into a functional whole, so selection on one trait can cause evolution to occur on another trait, sometimes with mildly maladaptive consequences.

## INTRODUCTION

Has human evolution stopped? Many evolutionary biologists have answered this question in the affirmative. For example, the distinguished paleontologist Stephen Jay Gould^1^ stated:

“There’s been no biological change in humans in 40,000 or 50,000 years. Everything we call culture and civilization we’ve built with the same body and brain”.

The basic rationale behind the conclusion that human evolution has stopped is that once the human lineage had achieved a sufficiently large brain and had developed a sufficiently sophisticated culture (sometime around 40,000–50,000 years ago according to Gould, but more commonly placed at 10,000 years ago with the development of agriculture), cultural evolution supplanted biological evolution. However, many evolutionary biologists have not accepted this argument, and indeed some have come to exactly the opposite conclusion. For example, Cochran and Harpending^2^ argue that “human evolution has accelerated in the past 10,000 years, rather than slowing or stopping, and is now happening about 100 times faster than its long-term average over the 6 million years of our existence.”

The answer to the question of whether or not human evolution has stopped has medical implications. To those who advocate that human evolution has stopped, modern medicine is just one more example of cultural evolution supplanting biological evolution. For example, advocates of human evolution having stopped believe that we no longer adapt to infectious diseases through natural selection; rather, we adapt culturally through the development of vaccines, antibiotics, and public health policies. Under this view, the common systemic diseases that we suffer from, such as type II diabetes, heart disease, etc., arise from our biological adaptations to a pre-agricultural environment that have persisted into the present because human evolution has stopped. Indeed, Armelagos^3^ has argued that human health has actually declined in many ways since the development of agriculture because we are stuck in bodies that are biologically adapted to a Stone Age environment.

In this review, I will argue that human evolution has not stopped, and our ongoing evolution has many medical and health implications. The rationale for the cessation of human evolution has three fundamental flaws. First, it is based on the premise that cultural evolution eliminates adaptive evolution via natural selection. However, all organisms adapt to their environment, and in humans much of our environment is defined by our culture. Hence, cultural change can actually spur on adaptive evolution in humans. The second flaw in the argument is the false premise that evolution is the same as adaptive evolution. Evolution is a change in the type or frequencies of genes or gene combinations in the gene pool over time, with the gene pool being the set of genes that are collectively shared by a reproducing population.^4^ Natural selection is a powerful mechanism for altering the frequencies of genes in the gene pool, but patterns of dispersal, system of mating, population size, and other factors can also cause alterations in the gene pool. Evolutionary change is determined not by one evolutionary mechanism operating in isolation, but rather by the several mechanisms interacting in concert.^4^ Human culture has dramatically changed the relative strengths of these other evolutionary mechanisms, once again spurring on much recent and ongoing human evolution. Third, traits are developmentally correlated, so that even a neutral trait can evolve due to selection on another trait. Hence, when cultural innovations weaken or eliminate natural selection on a trait, this alters the balance of evolutionary forces in a manner that induces further, albeit non-adaptive, evolutionary change in the neutral trait.

## ADAPTIVE EVOLUTION INDUCED BY HUMAN CULTURE

There is no doubt that agriculture and its continuing development has greatly altered the human environment. Environmental change often induces adaptive evolution, and humans are no exception. I will illustrate this first by examples of human evolution in response to infectious diseases. Agriculture altered the human environment in many ways, but two important alterations were in the numbers of humans and in their local population densities. Since the development of agriculture, the human population has grown in a roughly exponential fashion. Agriculture induces a more sedentary life style, and people need to live near their fields. As a result, even early agricultural systems resulted in large increases in local human densities. This combination of increased numbers of people and increased local densities created a new demographic environment that was ideal for the spread of infectious diseases. In this manner, agriculture increased the importance of infectious agents as selective factors in human evolution. A good example of this is seen in the pioneering work of Wiesenfeld.^5^ The Malaysian agricultural system, first developed in southeast Asia, makes extensive use of root and tree crops that are adapted to wet, tropical environments. The Malayo-Polynesian speaking peoples who developed this agricultural system also became excellent sailors who colonized many islands, including the island of Madagascar off the east coast of Africa around 2000 years ago. The Malaysian agricultural system was later taken up by Bantu speaking peoples on the African mainland about 1500 years ago and quickly spread throughout the wet, tropical sections of that continent. In the intact rainforests of Africa, malaria is a rare disease, but in those areas into which the Malaysian agriculture complex was introduced, malaria became common. The increased numbers and densities of people allowed more individuals to be infected at any given time and for infected individuals to be in close proximity to uninfected individuals, which in turn increased the probability of transmission of malaria via mosquitoes. Because of agriculture, malaria became a major infectious agent in this, and other, human populations, and hence a major selective agent. The result is that human populations began to adapt to malaria via natural selection. In sub-Saharan Africa, one of the main adaptations was by natural selection increasing the frequency of the sickle-cell allele at the hemoglobin β-chain locus, which confers resistance to malaria in individuals heterozygous for the sickle cell allele. Similar selective forces were introduced wherever agriculture created the conditions to make malaria a sustained, epidemic disease, and human populations in turn adapted to malaria by increasing the frequency of a number of alleles at many different loci, including the various thalassemias and glucose-6-phosphate-dehydrogenase deficiency alleles in addition to sickle cell.^4^ In terms of the numbers of people affected, these anti-malarial adaptations alone constitute the vast bulk of the classical Mendelian genetic diseases that afflict humanity. Other Mendelian genetic diseases have also been hypothesized to be selected as adaptations to human-created environments. For example, Ashkenazi Jewish populations have high frequencies of disease alleles at four different genetic loci – Tay-Sachs, Gaucher, mucolipidosis type IV, and Niemann-Pick – that all result in defects in sphingo-lipid storage. Motulsky^6^ hypothesized that all four of these genetic diseases represent adaptations to tuberculosis, which in turn became an important selective agent due to the formation of ghettoes, although this hypothesis remains controversial.^7^ Regardless, there is no doubt that most genetic disease in humans is due to natural selection adapting human populations to infectious agents whose selective importance was augmented, not diminished, by cultural evolution.^8^

Despite the advances in modern medicine, infectious agents remain an important selective agent in humans today. The scourge of malaria has not gone away, with 20,000 people dying every week from malaria.^9^ Moreover, as human populations have grown, we have altered our environment by intruding upon the habitats of more and more other species. The result has been that many infectious diseases of other species have ever-increasing chances to infect humans, and some of these cross-species infectious agents have successfully adapted to humans as their hosts. These culturally induced environmental changes have created a whole new area of health concern: emerging infectious diseases. One of the more dramatic recent examples has been the evolution of HIV from SIV, a retrovirus that infects other primates such as chimpanzees.^10^ The successful adaptation of HIV to humans has in turn created a selective force for humans to adapt to HIV, which we can actually observe in current human populations.^11^,^12^

As the above examples show, human cultural evolution did not stop human populations from adapting to infectious diseases but rather most likely intensified human adaptive evolution to infectious diseases. The same is also true for systemic diseases. Rather than being an evolutionary legacy from the stone age, there is much evidence that the genes underlying risk to many common systemic diseases were selected for their effects after the development of human agriculture. One of the more common systemic diseases plaguing humans today is type II diabetes, which is increasing at an alarming rate. This increase is so rapid that it cannot be due to evolutionary changes in the human population but rather to environmental changes, such as changes in diet and lifestyle.^13^ Nevertheless, type II diabetes, and many other systemic diseases, still can still reflect the impact of adaptive evolution in recent human history.

The idea that genes predisposing an individual to type II diabetes could represent recent adaptive evolution was first proposed by Neel^14^ as the “thrifty genotype hypothesis”. This hypothesis postulates that the same genetic states that predispose one to diabetes also result in a quick insulin trigger even when the phenotype of diabetes is not expressed. Such a quick trigger is advantageous when individuals suffer periodically from famines since it would minimize renal loss of glucose and result in more efficient food utilization. When food is more plentiful, selection against these genotypes would be mild because the age of onset of the diabetic phenotype is typically after most reproduction and because the high sugar, high calorie diets found in modern societies that help trigger the diabetic phenotype are very recent in human evolutionary history.

When Neel proposed this hypothesis, little was known about genetic factors that would predispose an individual to diabetes, but many genome-wide association studies have now identified several genetic loci that have such predisposing alleles.^15^ Moreover, there have now been multiple population surveys showing that the incidence of diabetes in a current high calorie dietary environment is higher in populations with a recent history of exposure to famines or calorie-restricted diets.^16^–^19^ For example, the Pima Indians of the American Southwest were formerly hunter-gatherers and farmers who used irrigation to raise a variety of groups, but principally maize. However, they were living in an arid part of the country, and their maize based agricultural system was subject to periodic failures during times of drought. This was accentuated in the late nineteenth century when European American immigrants diverted the headwaters of the rivers used by the Pimas for irrigation, resulting in widespread starvation. With the collapse of their agricultural system, the surviving Pimas were dependent on a government dispensed diet that consisted of high-fat, highly refined foods. Currently among adult Pima Indians, 37% of the men and 54% of the women suffer from type 2 diabetes, one of the highest incidences known in human populations.^19^ Another example is provided by the human population on the Micronesian island of Nauru.^17^,^18^ The Nauruans suffered from two extreme bouts of natural selection for thrifty genotypes in their recent history. First, their population was founded by people who undertook interisland canoe voyages lasting several weeks. In numerous attested examples of such lengthy canoe voyages, many voyagers died of starvation. Second, the Nauruans were then set apart from most other Pacific Islanders by their extreme starvation and mortality during the Second World War. Both of these episodes would have resulted in strong selection for thrifty genotypes. After World War II, an external mining company signed a lucrative deal with the Nauruans for the rights to phosphate-rich bird guano. With their newfound wealth, refined food became abundant. In this new dietary environment, some 28% of the adult population suffers from type 2 diabetes whereas in the previous generation diabetes was virtually unknown.

The observations summarized above support the thrifty genotype hypothesis, but perhaps the strongest evidence comes from the development of analytical methods that can detect the presence of recent positive selection for an allele by the signature such selection leaves behind in the genomic region around a selected variant. Several of the diabetes predisposing alleles have a significant signature of recent positive selection, particularly in those populations most susceptible to diabetes.^20^–^23^ These observations directly show that the genetic risk factors for diabetes have been favored by natural selection in recent human evolutionary history. Moreover, these same new analytical methods have revealed a large number of other genes that have been under intense positive selection in humans and that are related to recent cultural changes, particularly in agriculture.^24^

Interestingly, there is no compelling evidence to suggest that foraging and agricultural societies differ in either their frequency or severity of food shortages.^25^ However, the mathematical theory behind such sporadic selective episodes indicates that the elevation of the frequency of such predisposing alleles is strongest right after the food shortage and should decay over time.^26^ Consequently, stone age famines are unlikely explanations for the current high frequencies of these alleles. Moreover, stone age famines would not predict that observed pattern of these alleles being in highest frequencies in current populations that have been subjected to severe food shortages in the recent past. Unfortunately, the thrifty genotype hypothesis has often been portrayed as an example of past adaptation to a paleolithic lifestyle^25^,^27^ despite the fact that Neel, the originator of the hypothesis, used examples of populations subject to recent food shortages, such as the Pima Indians, as the primary support for the hypothesis.^19^ Hence, both observations and theory indicate that thrifty genotypes are present in current human populations as an adaptation to recent events and are *not* a legacy of human evolution having stopped in the paleolithic.

The thrifty genotype has been expanded and applied to the genetic risk factors predisposing individuals to many other common systemic diseases, such as coronary artery disease,^28^,^29^ metabolic syndrome,^27^ and hypertension.^27^ Thus, most of the common systemic disease in humans may well be frequent because of natural selection operating in recent, even historical, times. Our culture constitutes an environment that induces natural selection in humans. Adaptive evolution is therefore proceeding in modern human populations, and much of this recent human evolution bears directly upon the incidences of infectious, genetic, and systemic diseases in humans.

## RECENT HUMAN EVOLUTION DUE TO FACTORS OTHER THAN NATURAL SELECTION

The argument that human evolution has stopped is usually phrased in terms of cultural evolution supplanting adaptive evolution via natural selection. However, there are many factors that can cause evolution other than natural selection,^4^ but I will only mention three in this article: mutation, genetic drift, and gene flow. The evolutionary impact of these three evolutionary forces has been strongly altered in recent human history and continues to change rapidly, thereby causing an acceleration, not a diminution, of the rate of human evolutionary change, both non-adaptive and adaptive because these other evolutionary mechanisms also interact with natural selection.

As mentioned before, human population size has and continues to increase at a rapid rate since the development of agriculture. Mutations are the raw material of all evolutionary change. A small population will have very few new mutations at any given time. For example, suppose a specific nucleotide mutation has a probability of 10^−9^ of occurring per gene per generation at an autosomal locus. In a diploid population of 500, there are 1000 copies of an autosomal gene, so the expected number of new mutations to this specific form in any given generation is 10^−6^; that is, there is only one chance in a million of this mutation occurring any given generation. The human population size is now at 6.8 billion, so for an autosomal locus we would expect 13.6 occurrences of this specific mutation every generation. The large human population size is causing humans to enter into a very rare evolutionary zone that few organisms have ever reached; the zone in which virtually every single-step mutational change is possible in every generation. The retrovirus HIV-1 has achieved this evolutionary zone given its population sizes in the multiple billions within a single host and its high mutation rate, and this is the primary reason why HIV-1 is capable of such rapid and parallel evolution in different infected individuals.^30^

Such a massive reservoir of mutational variants in modern humans greatly augments the potential for all evolutionary change, including adaptive evolution through natural selection. Indeed, we can already see the evolutionary impact of this simple consequence of large population size. As mentioned in the previous section, the adoption of the Malaysian agricultural complex by Bantu-speaking peoples greatly altered the selective environment in wet, tropical Africa around 1500 years ago in such a way that natural selection now favored a specific nucleotide change from an A to a T in the middle position of the 6^th^ codon of the hemoglobin beta chain gene; that is, the sickle-cell allele. What is more remarkable is that this specific sickle-cell mutation went to high frequency multiple times in sub-Saharan Africa alone from four independent mutations of this specific nucleotide.^31^,^32^ The ability of large populations to produce a huge reservoir of mutational variants means that human populations are more evolutionarily responsive than ever to changes in the environment. This increased responsiveness is particularly important when dealing with temporary but intense selective forces, such as the sporadically occurring food shortages underlying the thrifty genotype hypothesis. As noted previously, there is no evidence that agriculture made food shortages more common or severe as a selective agent in human evolution, but agriculture did ensure that a famine affecting an agricultural population would be far more likely to result in selection for thrifty genotypes than a paleolithic famine simply because the chances of the mutations underlying thrifty genotypes being present in the population exposed to a famine would be orders of magnitude higher under agriculture than under a foraging culture. Thus, the thrifty genotypes that underlie so many of the common systemic diseases that afflict current human populations are much more likely to reflect recent human evolution under agriculture than adaptations to a paleolithic environment.

The evolutionary forces of genetic drift and gene flow will be discussed together, as they interact so strongly with one another. Genetic drift is the change in allele frequencies that is induced by sampling a finite number of genes in a population to produce the next generation. All finite populations evolve due to this random sampling error, with the strength of genetic drift being inversely proportional to the population size. Although genetic drift causes random fluctuations in allele frequencies, it has some very predictable properties. First, just by chance alone, an allele can randomly drift to a frequency of 0 or 1. When this happens, the genetic variation at this locus is lost. Hence, the smaller the population size, the greater genetic drift, and the more rapidly genetic variation is lost by random sampling processes. Second, when a species is split into multiple local populations, genetic drift causes random changes in allele frequencies in all of them. Because the changes are random, they are unlikely to be in the same direction in every local population. Hence, genetic drift leads to genetic differences between local populations, and the smaller the population size, the greater the expected differences among local populations.

Gene flow occurs when either individuals or gametes disperse from one local population to another through reproduction. Gene flow can introduce a mutation that arose in one local population into the gene pool of another local population. Hence, gene flow tends to increase the amount of genetic diversity found within local populations. The genetic interchange associated with gene flow also reduces the genetic differences among local populatons.^4^ Note that genetic drift and gene flow have exactly opposite effects on genetic variation within local populations (decreased by drift, increased by gene flow) and genetic differences among local populations (increased by drift, decreased by gene flow). As a result, the balance of genetic drift to gene flow is the primary determinant of how a species’ genetic variation is distributed within and among its local populations.

There is no doubt that the balance of genetic drift and gene flow has been greatly altered in recent human evolution and continues to change at a rapid pace. The increased human population size associated with the development of agriculture weakens the evolutionary force of genetic drift, and a wide variety of cultural innovations have greatly increased the ability of people to move across the globe and thereby augmented gene flow. Both of these alterations are increasing the level of genetic variation within local human populations and decreasing the genetic differences among human populations. This means that more and more of the genetic variation in the human gene pool exists at the level of individual heterozygosity (that is, the two copies of an autosomal gene borne by an individual are increasingly likely to be of different allelic states). This increased heterozygosity and switch to outbreeding because of enhanced dispersal has medical implications. It has long been known that inbreeding, which is fostered by having isolated local populations of small size, can have many deleterious effects on viability and health in general.^4^ This phenomenon is known as inbreeding depression and has been well documented in human populations,^33^,^34^ including increasing suspectibility to infectious diseases.^35^ In current human populations that still live in small, isolated populations, large contiguous stretches of the genome are homozygous (i.e. show no heterozygosity), but such genomic regions become increasingly rare and shorter in individuals sampled from developed countries that have larger population sizes and higher levels of dispersal from the birthplace.^36^

This rapid and ongoing shift to increased levels of heterozygosity in humans is already having discernable health effects. For example, Campbell et al.^37^ measured heterozygosity levels using 1184 genetic markers in four different Croatian populations that differed greatly in their degree of gene flow among local populations but that had similar diets, socio-economic status, and other factors. The levels of heterozygosity varied significantly among these four populations in the expected fashion. Several clinical traits were then regressed against relative heterozygosity, and all significant results indicated beneficial effects with increasing heterozygosity, as shown in Table 1. As heterozygosity levels continue to increase in humans due to our vastly increased abilities to disperse, these beneficial effects are expected to increase even more.

## NON-ADAPTIVE EVOLUTION INDUCED BY THE RELAXATION OF NATURAL SELECTION

The primary rationale for arguing that human evolution has stopped is that human culture has relaxed or even completely eliminated natural selection on certain traits. What is not generally appreciated is that the relaxation of selection on one trait can actually lead to its evolution by natural selection on other traits. All too often traits are regarded one-by-one, as if each trait could evolve independently of all other traits. However, the biological reality is that traits are correlated through developmental processes, pleiotropic genetic effects, and physiological connections. Consequently, it is commonplace that evolution of one trait induces correlated evolution on another trait. If the nature of these inherent correlations are known or estimated, then one test for natural selection on a set of traits is that they violate these inherent correlations over evolutionary time. If one trait is evolving due to natural selection, but a second trait is no longer being selected, selection on the first trait is expected to cause evolutionary change at the second trait in a manner consistent with the inherent correlations.

For example, there is no controversy that the human lineage has been strongly selected for increased brain size over the past 2 million years,^38^ and that one of the primary driving forces for this evolution of brain size has been our increasing use of learned culture as a means of dealing with the environment and social interactions. As the cultural sophistication of the human lineage increased, it perhaps did indeed reduce or eliminate selection on some traits. For example, most animals adapt to their diet through their teeth and jaws, but humans increasingly used tools and fire to prepare their food, thereby reducing the importance of jaw and tooth evolution as a means of adapting to the dietary environment.

**Figure 1:**
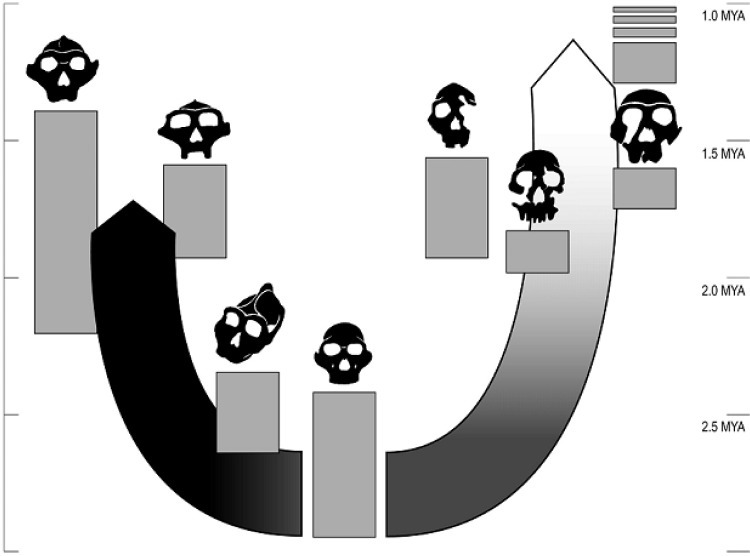
Natural selection on facial characteristics and diversity in early human evolution are shown in a temporal context. The two arrows indicate the robust austra-lopithecine lineage on the left and the lineage leading to modern humans on the right. The darker the shading, the more intense the selection on facial features. Reprinted from Ackermann and Cheverud^39^ with permission. Copyright (2004) National Academy of Sciences, USA.

Ackerman and Cheverud^39^ tested the hypotheses of selected versus neutral evolution of human teeth and jaws by comparing various hominid fossil measurements to the expected correlations among relative brain size, tooth size, and jaw size as inferred from modern-day humans, chimpanzees and gorillas, which all have remarkably similar developmental correlations for these traits. The results are shown in Figure 1. At the base of this figure is a skull of a gracile australopithecine, and stemming off that ancestral form are two lineages. The lineage on the left represents the robust australopithecines, and the lineage on the right is the one that led to modern humans. The arrows indicating the lineages are shaded to indicate the strength of the estimated selection on the face (mostly teeth and jaws measurements), such that the darker the shading, the more intense the selection. As can be seen, the robust australopithecine lineage was subject to very intense natural selection on their faces, indicating that they primarily adapted to their dietary environment through adaptive evolution of the teeth and jaws. In contrast, in the lineage leading to modern humans, the intensity of selection on the face diminishes with time, and by 1.5 million years ago there is no longer any detectable selection on human teeth and jaws. Ackerman and Cheverud^39^ interpreted this as being consistent with the hypothesis that cultural evolution in the human lineage had indeed eliminated natural selection on human teeth and jaws. However, this does *not* mean that human teeth and jaws have not evolved over the last 1.5 million years. During the last 1.5 million years, there was a large increase in brain size in the human lineage driven by natural selection, and given the developmental constraints common to humans, chimpanzees, and gorillas, human jaws and teeth would continue to evolve as a correlated effect of brain size evolution. In particular, jaws and teeth were predicted to become relatively smaller for our body size as a correlated response to increased brain size, with the jaw becoming relatively smaller more rapidly than the teeth. Hence, the elimination of natural selection directly upon teeth and jaws did not eliminate evolution on these traits because of natural selection for increased brain size. The result of this correlated evolution is that humans have a small, flat face compared to chimpanzees and gorillas, and that our jaws tend to be too small for our teeth, thereby giving rise to the modern profession of orthodontics.

## CONCLUSIONS

Has human evolution stopped? The answer is a definitive no. The only way to truly stop any biological organism from evolving is extinction. Evolution can be slowed by reducing and keeping population size to a small number of individuals. This will lead to a loss of most genetic variation through genetic drift and minimize the input of new mutations into the population. Since genetic variation is the raw material of all evolutionary change, prolonged small population size can severely diminish, but not completely eliminate, the ability of a population to evolve. However, this is certainly not the situation with humans. Our population size has been increasing over the last 10,000 years, and is now so large that the current human gene pool contains an immense reservoir of genetic variation. Hence, our evolutionary potential has never been higher. This genetic variation has indeed been utilized by episodes of recent, positive selection induced, not diminished, by cultural evolution. Our evolution is further driven by a radical change in the balance of genetic drift and gene flow that is rapidly causing a major evolutionary change in the human species in how genetic variation is distributed within and among local populations. Even when our cultural innovations do eliminate selection on a trait, that trait can still evolve as a correlated response to evolution of another trait, often in a non-adaptive fashion and sometimes in a mildly mal-adaptive fashion. As long as humans persist as a reproducing population, humans will evolve. This has been the lesson of the past 10,000 years, and is certainly what we can expect to continue for as long as our species persists on the Earth.

## Figures and Tables

**Table 1:** Statistically significant results of regressions of several clinical traits against heterozygosity levels in four Croatian populations. Modified from Campbell et al.^37^

**Trait**	**Sample Size**	**Regression Coefficient**	**Standard Error**	**Probability Level**
Systolic Blood Pressure	223	−102.8	42.0	0.015
Diastolic Blood Pressure	223	−47.7	20.5	0.021
Log(Total Cholesterol)	200	−1.083	0.439	0.014
Log(LDL Cholesterol)	201	−1.539	0.597	0.011
Forced Expiratory Flow_25_	200	−3.174	1.366	0.021
